# Prediction of I–V Characteristic Curve for Photovoltaic Modules Based on Convolutional Neural Network

**DOI:** 10.3390/s20072119

**Published:** 2020-04-09

**Authors:** Jie Li, Runran Li, Yuanjie Jia, Zhixin Zhang

**Affiliations:** School of Electronics and Control Engineering, Chang’an University, Xi’an 710064, Shaanxi, China; 2019132044@chd.edu.cn (R.L.); 2019232025@chd.edu.cn (Y.J.); 2019232014@chd.edu.cn (Z.Z.)

**Keywords:** photovoltaic module, convolutional neural network, multilayer perceptron, current–voltage curve

## Abstract

Photovoltaic (PV) modules are exposed to the outside, which is affected by radiation, the temperature of the PV module back-surface, relative humidity, atmospheric pressure and other factors, which makes it difficult to test and analyze the performance of photovoltaic modules. Traditionally, the equivalent circuit method is used to analyze the performance of PV modules, but there are large errors. In this paper—based on machine learning methods and large amounts of photovoltaic test data—convolutional neural network (CNN) and multilayer perceptron (MLP) neural network models are established to predict the I–V curve of photovoltaic modules. Furthermore, the accuracy and the fitting degree of these methods for current–voltage (I–V) curve prediction are compared in detail. The results show that the prediction accuracy of the CNN and MLP neural network model is significantly better than that of the traditional equivalent circuit models. Compared with MLP models, the CNN model has better accuracy and fitting degree. In addition, the error distribution concentration of CNN has better robustness and the pre-test curve is smoother and has better nonlinear segment fitting effects. Thus, the CNN is superior to MLP model and the traditional equivalent circuit model in complex climate conditions. CNN is a high-confidence method to predict the performance of PV modules.

## 1. Introduction

As well known, the problem of energy shortage in the world becomes serious. Solar energy has become an important new energy because of its cleanness and sustainability. According to the report of the International Energy Agency (IEA), the installed number of photovoltaic (PV) has been growing at a rapid speed in recent years [1]. As basic energy collection component of power generation system, the accurate and reliable modeling of PV module is quite significant in design, optimized simulation, operation and evaluation of photovoltaic power generation system [2,3,4]. The accurate results of model output could be used to get system information like maximum power point tracking (MPPT), power prediction [5,6,7,8,9,10,11]. In addition, some researchers suggest that the random forest (RF) ensemble learning algorithm and the emerging kernel based extreme learning machine (KELM) are explored for the detection and diagnosis of PV arrays early faults (including line-line faults, degradation, open circuit and partial shading). They are also based on the accurate acquisition of current–voltage (I–V) curve [12,13].

The I–V curve is an important index to characterize the performance of PV modules. It is proposed that accurate, efficient and reliable parameter extraction of solar photovoltaic (PV) models from the measured current–voltage (I–V) characteristic curves is important for evaluation, modeling and diagnosis of the actual operating state of in situ PV arrays [14]. Nowadays, manufacturers of photovoltaic modules provide standard reporting condition (SRC) or standard test condition (STC) ratings of PV modules. The main test conditions of PV modules are mainly under laboratory environment, which include irradiation intensity of 1000 W/m^2^, spectral distribution in accordance with the AM1.5 spectrum and temperature of 25 ± 1 °C for the PV module. However, in the practical engineering environment (outdoor weather conditions), these conditions rarely appear at the same time. The adequacy and applicability of PV modules under STC is still a controversial issue [15]. Actually, the PV modules based on SRC are still unreasonable for the real-world weather conditions. Therefore, performance test of PV module based on STC condition needs to be improved in the outdoor weather conditions [16]. Although the outdoor characteristics of PV modules can be predicted by algebraic or numerical methods, these methods used in photovoltaic system engineering ignore some second-order effects, like wind speed, shunt resistance, parasitic capacitance, spectral effect and non-linearity under low illumination, thus the prediction error of these methods is large [17]. Based on this, many photovoltaic modeling methods have been proposed in recent years. Generally, these methods can be divided into two types: the white box model based on equivalent circuit and the black box model based on regression [18,19,20]. The equivalent circuit method is used for the prediction of photovoltaic characteristics traditionally, in which the photovoltaic will convert into equivalent circuit. Through the analysis of the circuit, the mathematical expression of photovoltaic I–V characteristics is obtained. Some scholars have proposed a variety of equivalent circuit models of solar cell PV module, and carried out parameter identification according to the I–V characteristic data [21]. Accordingly, some researchers have proposed various parameter estimation techniques, including analysis methods and numerical methods [22,23,24,25]. Curve fitting is used in most of these methods, but only for obtaining equivalent circuit parameters. Thus, the accuracy of these methods is low, and it is difficult to obtain the important parameters by using these methods.

In recent years, the emergence of neural networks based on machine learning attracts more attention on characteristic prediction for PV module. Compared with the photovoltaic modeling technology based on the white box model, the photovoltaic modeling technology based on the data-driven black box model directly uses the regression method to build the PV model from the measurement data without any assumptions and constant measurements. Therefore, the disadvantages of the white box model could be avoided. The black box model is more accurate and versatile [26]. Normally, artificial neural network (ANN) based black box models include generalized regression neural network (GRNN), multilayer perceptron (MLP) neural network, etc. [27,28]. Although the above neural network model has achieved a positive effect in other fields, due to the strong uncertainty and environmental sensitivity of the PV module, the generalization performance and nonlinear fitting ability of these models in the PV performance prediction is needs to be improved and investigated.

At present, as a new black box model, convolutional neural networks (CNN) performs excellently in some scenes with features of multi-input multi-output (MIMO), strong nonlinearity and coupling, such as image processing, traffic flow prediction, etc. Therefore, CNN is expected to become a new method for photovoltaic performance prediction. A high confidence model of PV module is built based on CNN. Then the MLP and the traditional equivalent circuit method have been compared to investigate performance of the presented CNN model. Furthermore, this study solves the problem that it is difficult to obtain the solar spectral information under outdoor conditions pointed out by scholars such as Piliougine et al. [29], the laboratory STC conditions are replaced by the specific environmental conditions, such as irradiance, atmospheric pressure, humidity, temperature of the PV module back-surface, etc.

## 2. Experimental Equipment and Data Acquisition

In the practical engineering environment, the equipment operation is normally not stable due to the influence of light, wind and other factors. Thus, the experimental data obtained in the laboratory standard test environment (STC) cannot meet the requirement of real applications. It should be recognized that the modeling accuracy in the practical engineering environment are more important than in test environment. Therefore, an outdoor test platform is setup by National Renewable Energy Laboratory (NREL) in Coco, Florida, USA. This platform with an installation angle of 28.5 degrees and facing south direction is located on the peninsula southeast coast. The collected data include short-circuit current, maximum power, current and voltage of PV module in amperes when operating at maximum power, open-circuit voltage, irradiance, temperature of the PV module back-surface, ambient temperature, atmospheric pressure, relative humidity et al. In addition, there are 100 groups of current and voltage of I–V curve. The Xsi12922 [15] (Single-crystalline silicon) solar cell is used to do the test in this study.

Under the above conditions, data sets that NREL provides contain five factors, such as irradiance (*G*), temperature of the PV module back-surface (*T*_1_), ambient temperature (*T*_2_), relative humidity (*H*_a_) and atmospheric pressure (*P*_a_), these factors have a significant impact on the performance of PV modules [21,22,23,24,25]. Thus, performance of PV modules is tested according to these factors. In order to obtain the performance index of the Xsi12922 PV module directly, eight groups of working conditions are randomly selected for research in this study. The eight groups of working conditions are shown in Table 1.

**Figure 1:**
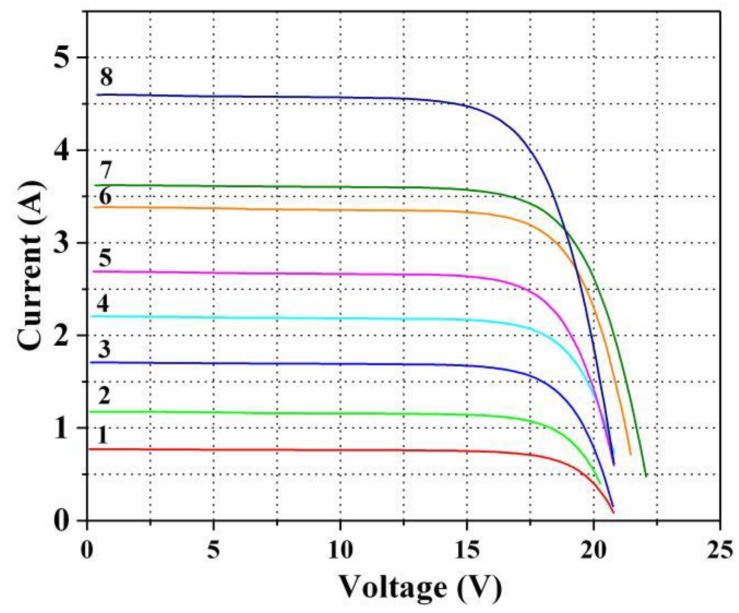
I–V curves of this PV module corresponding to the eight working conditions. Curve of 1–8 in the Figure correspond to the eight working conditions shown in Table 1, respectively.

The I–V curves of this PV module corresponding to the eight working conditions are shown in Figure 1.

## 3. Models Building and Training

### 3.1. MLP Model Establishment

MLP is used in ANN and the first layer of MLP is the input layer, the middle layer is the hidden layer and the last layer is the output layer. Connection mode between these layers is full connection. Assuming that the input layer is **X_i_** = [**x_1_**,**x_2_**,**x_3_**,…,**x_n_**], the weight matrix of the hidden layer is **W_i_** = [**w_1_**,**w_2_**,**w_3_**,…,**w_q_**] and the output layer is **Y_i_** = [**y_1_**,**y_2_**_,_**y_3_**,…,**y_n_**]. Set *f*(⋅) as the activation function and **x_n_**∈R^1*n^, **w_n_**∈R^n*1^, **b**∈R^q*n^. Thus, the forward propagation mode from the input layer to the output layer is [30]

(1) $$y=f(\sum_{i=1}^{n}w_{1}^{\left(m\right)}x_{i}+b_{1}^{\left(m\right)}),m=1,2,3,\ldots ,n$$

Decompose (1) into a single neuron matrix

(2) $$y=f\left(\left[\begin{array}{c} \begin{array}{c} \begin{array}{c} x_{1}^{T}\\ x_{2}^{T} \end{array}\\ \ldots \end{array}\\ x_{n}^{T} \end{array}\right]\left[w_{1},w_{2},\ldots ,w_{q}\right]+b\right)$$

In this study, Adam algorithm is used to optimize the backward propagation of MLP. Adam is a first-order optimization algorithm that improves the traditional random gradient descent (SGD) algorithm, which can deal with the noise sample and sparse gradient and has a natural annealing effect [31,32,33].

### 3.2. CNN Model Establishment

**Figure 2:**
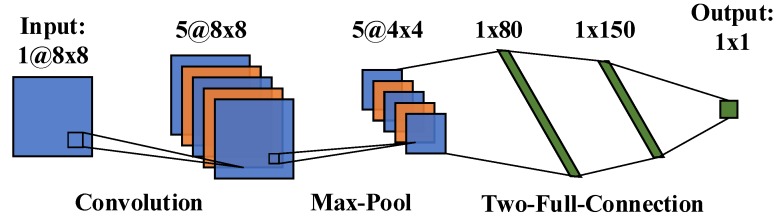
Structure of the convolution neural network.

CNN is a kind of feed-forward neural network; it has been used in face recognition, target detection, speech recognition, optical character recognition, etc. [34,35,36]. Then it is applied to regression analysis in this study. The artificial neuron of CNN can feel the local characteristics of the sample. The features of the current layer and the next layer of CNN can be synthetically connected to achieve the effect of feeling the whole sample and each part of the sample is analyzed in this way, thus the prediction error can be reduced. A single channel CNN model is designed in this study and shown as Figure 2. It is composed of five layers, namely general input layer, convolution layer, pooling layer and full connection layer (it has two layers). Therefore, higher-level convolution neural network is evolving based on this structure.

Input of the l layer in convolution layer is **x**∈R_l_^W×H×D^ in the three-dimensional case. Set the convolution kernel of the l layer as **m**∈R_l_^W×H×D^, then the number of the convolution kernel is *D* and the output dimension is 1 × 1 × 1 × 1 × *D* at the same location. Thus, *D* is the number of channels of layer *l* + 1 characterized by *x^l^*^+1^ and the convolution operation channel number of *D^l^*^+1^ is

(3) $$y_{i^{l+1},\;j^{l+1},\;d}=\sum_{i=0}^{W}\sum_{j=0}^{H}\sum_{d^{l}=0}^{D}f_{i,j,d^{l},d}\times x_{i^{l+1}+i,j^{l+1}+j,d^{l}}^{l}$$

where, the term of *f* indicates the weight matrix in CNN and (*i*^l+1^, *j*^l+1^) is the convolution coordinate, which should meet

(4) $$0\leq i^{l+1}<H^{l}-H+1=H^{l+1}$$

(5) $$0\leq j^{l+1}<W^{l}-W+1=W^{l+1}$$

In this study, the input data can be regarded as a two-dimensional 8 × 8 matrix structure and 3 × 3 convolution kernels is adopted. It can be seen that the weight matrix is the same for all inputs at different positions, which is the feature of weight sharing in convolution. In addition, a bias term can be added and in the back propagation, the learning rate of random gradient drop can be set for the weight and paranoia of this layer. If it is required, the bias of a certain position of a certain layer can also be set to zero.

Set the size of the input data volume as *W*_1_ × *H*_1_ × *D*_1_, the output size as *W*_2_ × *H*_2_ × *D*_2_, the number of convolution kernels as *K*, the space size of convolution kernels as *f*, the step size as *s* and the edge filling amount as *p*, thus the input and output shapes meet the following equation

(6) $$w_{2}=(w_{1}-f+2p)/s+1$$

(7) $$h_{2}=(h_{1}-f+2p)/s+1$$

(8) $$d_{2}=k$$

Average pooling and maximum pooling are the basic operations in the pooling layer and can be described as (9) and (10)

(9) $$y_{i^{l+1},\;j^{l+1},d}=\frac{1}{HW}\sum_{0\leq i<H,0\leq j<W}x_{i^{l+1}+i\times H+i,j^{l+1}\times W+j,d^{l}}^{l}$$

(10) $$y_{i^{l+1},j^{l+1},d}=\underset{0\leq i<H,0\leq j<W}{\mathrm{MAX}}x_{i^{l+1}+i\times H+i,j^{l+1}\times W+j+j,d^{l}}^{l}$$

The first layer of CNN is pool as **p**^l^∈R_l_^W×H×D^, then the pooling results of each layer cover the original results.

The core space of pooling is 2 × 2 matrix and the input data size is *W*_2_ × *H*_2_ × *D*_2_, the output size is *W*_3_ × *H*_3_ × *D*_3_, size of the core space is *f*, the step size is *s*, then the shape of the input and the output meet the following equation

(11) $$w_{3}=(w_{2}-f)/s+1$$

(12) $$h_{3}=(h_{2}-f)/s+1$$

(13) $$d_{3}=d_{2}$$

The neurons of CNN in the full connection layer are all connected to the activation data in the previous layer, and Adam algorithm is also used to optimize the backward propagation of CNN [31,32].

### 3.3. Model Training

**Figure 3:**
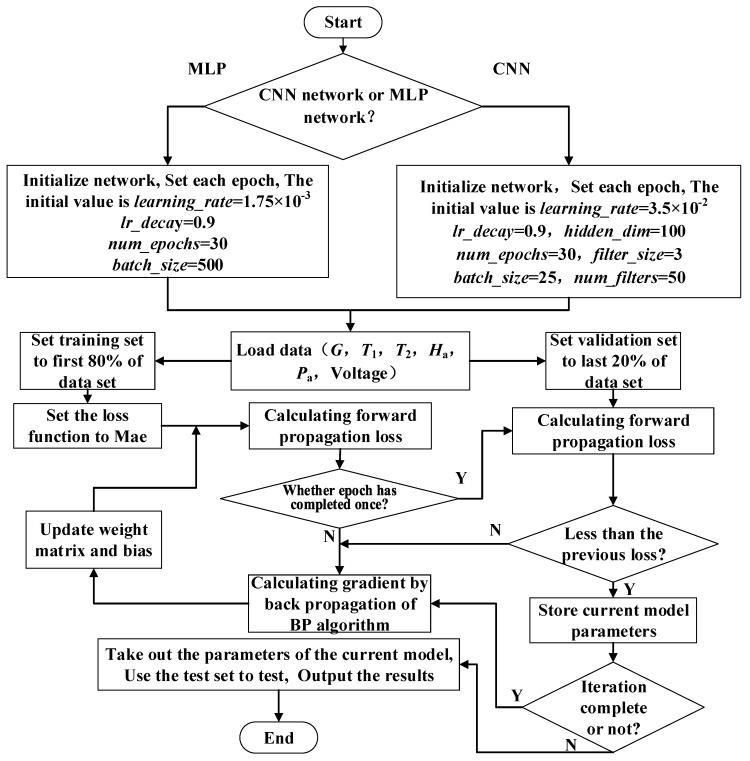
Program flow chart of multilayer perceptron (MLP) and convolutional neural network (CNN).

The ANN and CNN model presented are used to predict the I–V curve, and the voltage and five environmental conditions described above are adopted as input conditions. The loss function for MLP and CNN model is defined as *MAE*

(14) $$MAE=\frac{1}{N}\sum_{i=1}^{N}\left|y_{i}^{\prime }-y_{i}\right|$$

where, *N* is the total data set from NREL, *y*’_i_ is the predicted data, *y*_i_ is the measurement data. Actually, *MAE* is the average absolute error. Back propagation (BP) algorithm is used for the model optimization in this study. The training process of MLP and CNN is illustrated in Figure 3.

Remove missing data items and randomly and uniformly select 5000 sets of data with irradiance between *G* = 150 W/m^2^–1000 W/m² and temperature between 15–40 °C, and select the first 90% as the training set and the last 10% as the validation set. Furthermore, randomly and uniformly select 2000 sets of data with irradiance between *G* = 150 W/m^2^–1000 W/m² and temperature between 15–40 °C as the test set.

Two layers of MLP structure are used and the activation function is set as LeakyRelu. In the MLP model, the learning rate is set as 1.75 × 10^-3^, the learning decay is 0.9, the training generation is 30, the small batch size is 500, the first layer of neurons is set as 150 and the second layer of neurons is set as 100.

Two layers of CNN structure are used, and the activation function is also set as LeakyRelu. In the CNN model, the learning rate is set as 3.5 × 10^-4^, the learning decay is 0.9, the training generation is 30, the small batch size is 25, the convolution kernel dimension is 50, the convolution kernel size is 3 and the hidden layer dimension is 100.

**Figure 4:**
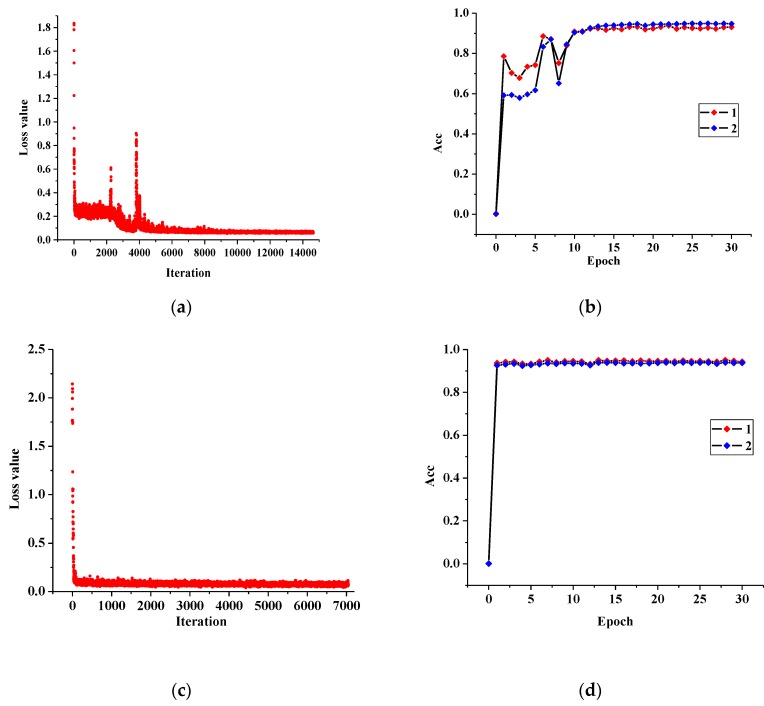
Model training process of MLP and CNN. (**a**) Loss function of MLP; (**b**) *Acc* of MLP; (**c**) Loss function of CNN; (**d**) *Acc* of CNN.

MLP and CNN models are trained according to the program flow as shown in Figure 3. The training process is illustrated in Figure 4. Figure 4a,b indicate the changes of loss function and accuracy in MLP training while Figure 4c,d indicate the changes of loss function and accuracy in CNN training. In Figure 4, *Acc* is defined as

(15) $$Acc=1-\frac{\left|y_{i}^{\prime }-y_{i}\right|}{y}$$

Curves 1 and 2 in Figure 4b,d show the accuracy in training set and test set respectively. It can be seen from Figure 4 that the CNN model has a fast convergence rate and a stable process while the MLP model has a relatively slow convergence rate and has a series of oscillating links. However, the final convergence values of the two models are similar.

## 4. Results Analysis

### 4.1. The Accuracy Analysis

**Figure 5:**
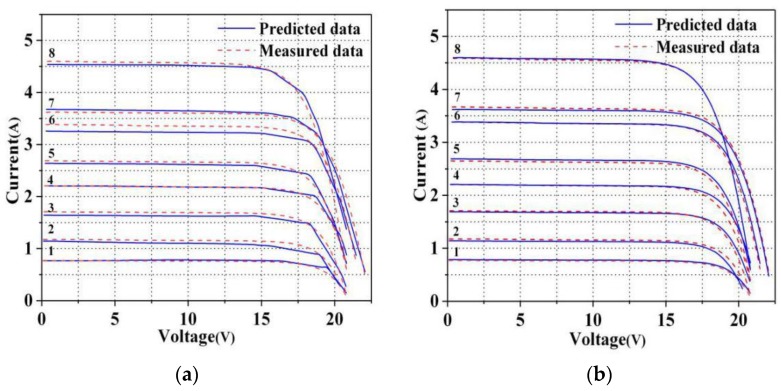
The fitting effect of I–V curves. (**a**) Linear fitting effect of MLP mode; (**b**) Linear fitting effect of CNN model.

MLP and CNN are trained to obtain the optimal training models. After the test set verification of the optimal training model, the results obtained are shown in Figure 5. Figure 5a,b indicate the linear fitting effect of I–V curves predicted by MLP model and CNN model. Compared with MLP, there are fewer cusps in the nonlinear fitting curves of CNN model, thus it shows that the fitting effect of CNN model is better than that of MLP model. The cusps will greatly reduce the prediction accuracy and increase the error and the degree of difficulty in finding the maximum power for PV modules.

The evaluation terms for the I–V curves are defined as Mean Absolute Error (MAE, seen in equation (14)) and Root Mean Square Error (RMSE) and *RMSE* is

(16) $$RMSE=\sqrt{\frac{1}{N}\sum_{i=1}^{N}{\left(y_{i}^{\prime }-y_{i}\right)}^{2}}$$

where, *N* is set as 50 in this study. *MAE* shows the distance between the predicted value and the measured value, which is close to zero in ideal situations, while *RMSE* further demonstrates the degree of dispersion between the predicted value and the measured value. Obviously, smaller *RMSE* means higher aggregation of the errors. *RMSE* and *MAE* of the eight groups of I–V curves are shown in Table 2.

It can be seen from Table 2 that, when the irradiation intensity is lower than 500 W/m^2^, *MAE* and *RMSE* of MLP and CNN are relatively low, while *MAE* and *RMSE* of MLP become high when he irradiation intensity is higher than 500 W/m^2^.Thus, accuracy of the CNN model is obviously higher than the MLP model. In addition, it indicates that the aggregation of the errors of CNN is higher than that of MLP.

### 4.2. Analysis of the Fitting Degree

The fitting degree of the I–V curves is analyzed in the following. As we all know, Euclidean distance is the distance between two points in a metric space. Now we define the distance from the point to the line as

(17) $$\rho =\frac{\left|Ax+By+c\right|}{\sqrt{A^{2}+B^{2}}}$$

**Figure 6:**
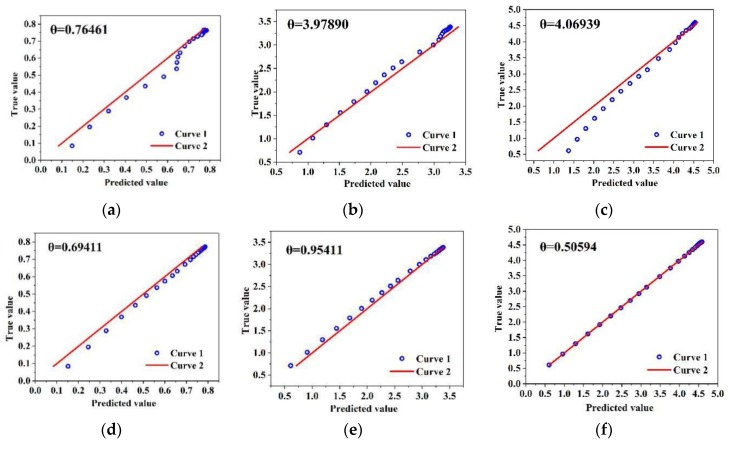
Analysis of the fitting degree for MLP and CNN models. (**a**) MLP model at *G* = 153.7 W/m^2^; (**b**) MLP model at *G* = 653.4 W/m^2^; (**c**) MLP model at *G* = 909.0 W/m^2^; (**d**) CNN model at *G* = 153.7 W/m^2^; (**e**) CNN model at *G* = 653.4 W/m^2^; (**f**) CNN model at *G* = 909.0 W/m^2^.

All distances are accumulated as

(18) $$\theta =\sum_{i=1}^{n}\frac{\left|Ax_{i}+By_{i}+c\right|}{\sqrt{A^{2}+B^{2}}}$$

where, *x*_i_ can be regarded as predicted value, *y*_i_ can be regarded as measured value. If *x*_i_ equaled *y*_i_, there would be a line like *y* = *x*. Ideally, the slope of this line is one. In fact, the predicted and measured values are scattered around the line, so it is best for *θ* to be close zero. In this study, the predicted value and the measured value are mapped in the same coordinate system and the analysis of the fitting degree is shown in Figure 6. Curve 1 and curve 2 in Figure 6 indicate the data points and the line like *y*=*x*.

Analysis of the fitting degree is researched by randomly selecting three group of working conditions in the test set. Figure 6a–c show the *θ* of MLP model, while Figure 6d–f show the fitting degree of CNN model. *θ* is 0.76461, 3.97890 and 4.06939 in (a), (b) and (c), respectively, while *θ* is 0.69411, 0.95441 and 0.50594 in (d), (e) and (f). It can be concluded that *θ* of the CNN model are closer to zero when compared to the MLP model. Thus, the fitting degree of the CNN model is obviously higher than the MLP model.

**Figure 7:**
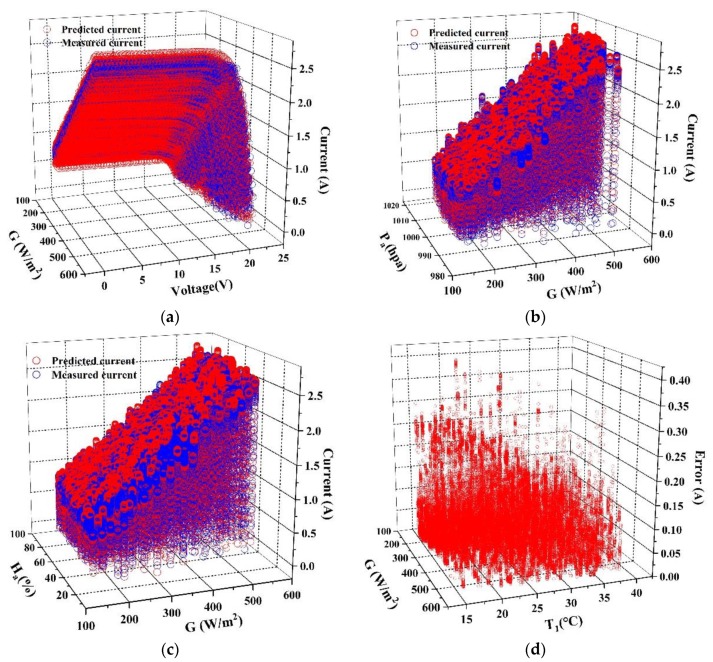
Analysis of the predicted value form MLP mode. (**a**) Changes of the current with *G* and *V*; (**b**) Changes of the current with *G* and *P*_a_; (**c**) Changes of the current with *G* and *H*_a_; (**d**) Changes of the error with *G* and *T*_1_.

**Figure 8:**
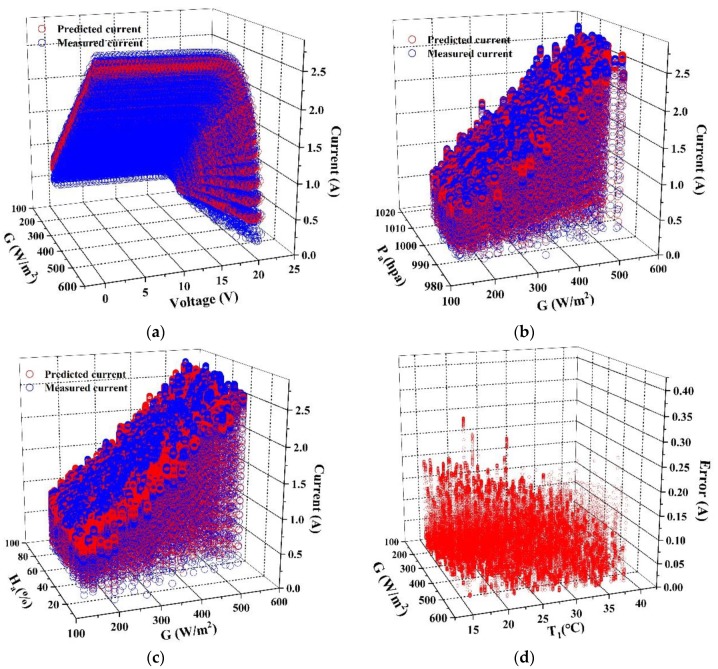
Analysis of the predicted value form CNN model. (**a**) Changes of the current with *G* and *V*; (**b**) Changes of the current with *G* and *P*_a_; (**c)** Changes of the current with *G* and *H*_a_; (**d**) Changes of the error with *G* and *T*_1_.

A large-scale analysis for CNN and MLP models are performed and the results are shown as Figure 7 and Figure 8. Figure 7 and Figure 8 exhibit the predicted current from MLP and CNN models under the same conditions. The error in (d) of Figure 7 and Figure 8 is defined as *y*’_i_-*y*_i_.

Figure 7 and Figure 8 show the predicted value from both MLP and CNN models with different input variables. The irradiance (*G*), voltage (*V*), temperature of the back-surface (*T*_1_), relative humidity (*H*_a_) and atmospheric pressure (*P*_a_) are chosen as input variables, then the comparison between the predicted and the measured value is shown in Figure 7a–c and Figure 8a–c respectively. Figure 7d and Figure 8d show the predicted errors of the current changed with *G* and *T*_1_. It can be calculated from Figure 7a–c and Figure 8a–c that, *MAE* of MLP and CNN models is 0.0671 and 0.0307 respectively and compared with MLP, CNN shows better following ability in current prediction.

By analyzing the data in Figure 7d and Figure 8d, 90% of MLP prediction error are range in 0–0.175, while 90% of CNN prediction error are range in 0–0.03. The analysis above show that the robustness and accuracy of CNN model are much higher than MLP model in I–V curves prediction for PV module.

### 4.3. Comparison with Equivalent Circuit Method

Some scholars have published the calculation of I–V curve by traditional equivalent circuit and have done more in-depth research [37]. However, the accuracy of the equivalent circuit and its feasibility are still unsatisfactory. The MLP model is also compared it with traditional equivalent circuit model in detail and it is pointed out that the black box model is a simplified mathematical model and the accuracy of MLP model is higher than the equivalent circuit model under the condition of *RMSE* and *MAE* evaluation [15,21]. Thus, the black box model is investigated to further improve its accuracy. Then the black box model used in this comparative study is described as follows [15,21].

The equivalent circuit equation of the single-diode model for a solar cell is given [21].

(19) $$I=I_{pv}-I_{0}\left[\exp\left(\frac{q(V+IR_{s})}{nKT}-1\right)-\frac{V+IR_{s}}{R_{sh}}\right]$$

This is the basic equation. In addition, we should also determine the transformation between parameters under standard test conditions and parameters under operating environment [21].

(20) $$I_{pv}=\left[I_{pv,STC}+\alpha \left(T-T_{STC}\right)\right]\cdot \frac{G}{G_{STC}}$$

(21) $$I_{0}=I_{o,STC}{\left(\frac{T}{T_{STC}}\right)}^{3}\exp\left[\frac{1}{K}\left(\frac{E_{g}}{T_{STC}}-\frac{E_{g}}{T}\right)\right]$$

(22) $$R_{s}=R_{s,STC}\cdot \frac{T}{T_{STC}}\cdot \left[1-\beta \cdot \ln\left(\frac{G}{G_{STC}}\right)\right]$$

(23) $$R_{sh}=R_{sh,STC}\cdot \frac{G_{STC}}{G}$$

(24) $$n=n_{STC}\cdot \frac{T}{T_{STC}}$$

(25) $$E_{g,T}=E_{g,STC}\cdot \left[1-0.0002677\left(T-T_{STC}\right)\right]$$

where the *I*_pv_ is the photocurrent and *I*_o_ is the saturation current of the diodes; Constant *q* is absolute value of electric charge of an electron (1.60217646 × 10^−19^ C); *K* is the Boltzmann constant (1.380653 × 10^−23^ J/K); *T* is the absolute temperature of the PV cell; *n* denotes the ideal factor of the diode; *R*_s_ is the equivalent series resistance and *R*_sh_ is the equivalent parallel resistance [21]. the *I*_pv,STC_, *I*_o,STC_, *R*_s,STC_, *R*_sh,STC_ and *n*_STC_ are values of the five unknown parameters at the STC [21]. Where the *α* is temperature coefficient of the photocurrent; The *β* represents the irradiance coefficient of the series resistance; The *E*_g_ is the energy band gap and is equal to 1.121 eV for silicon solar cell at STC.

According to the equivalent circuit method shown in reference 21, there are 2000 test sets selected above calculating the I–V curve corresponding to each group of conditions. Then calculate the corresponding *RMSE* and *MAE*. Part of the results of the equivalent circuit are shown in Table 3. The results are compared with that of the MLP and the CNN models, as shown in Table 4.

From above research results it can be concluded that, the prediction level of MLP and CNN based on the black box model is significantly higher than that of the traditional equivalent circuit model. Furthermore, the prediction accuracy of CNN model is obviously higher than the MLP model. The *RMSE* of the equivalent circuit model is about ten times higher than CNN model through the calculation of 2000 sets of data shown in Table 4. Although MLP network has some improvement, its *RMSE* is about twice higher than CNN model.

## 5. Conclusions

CNN model is used to predict I–V curve of silicon PV module with consideration of irradiance, temperature of the back-surface, ambient temperature, relative humidity and atmospheric pressure in the practical engineering environment instead of STC laboratory conditions. In this way, a high confidence model of Xsi12922 PV module is built. In this paper, we mainly compare two neural network models and compare them with the traditional equivalent circuit model. *RMSE*, *MAE* and regression analysis are used for large-scale evaluation and tabulation. The results show that the accuracy of CNN model is higher than that of MLP model and much higher than that of equivalent circuit model.

The advantage of CNN model is that it is based on black box model and simplifies the traditional model. Therefore, the current value can be estimated accurately only under environmental conditions without any constant measurement. At the same time, its accuracy is better than the MLP model which is the same as the black box model. In this study, the presented CNN model shows high robustness and accuracy in the I–V curve prediction for PV module, which makes it superior to the traditional model and MLP model in the application of complex climate conditions.

## Figures and Tables

**Table 1:** The selected working conditions.

Working Conditions	Irradiance *G* (W/m^2^)	Temperature of PV Module Back-Surface *T*_1_ (°C)	Ambient Temperature *T*_2_ (°C)	Relative Humidity *H*_a_ (%)	Atmospheric Pressure *P*_a_ (hPa)
1	153.7	16.8	21.9	83.2	1002.9
2	237.5	23.3	24.6	42.3	1001.4
3	328.7	26.9	24.7	69.7	997.1
4	445.5	22.7	24.8	58.4	1007.6
5	537.9	28.2	24.3	59.0	1006.5
6	653.4	22.8	22.1	88.3	1007.9
7	726.9	19.5	23.4	73.8	1001.8
8	909.0	36.2	25.7	71.1	1015.1

**Table 2:** *RMSE* and *MAE* of I–V curves.

Working Conditions	Irradiance *G* (W/m^2^)	Algorithms	*MAE* (%)	*RMSE* (%)
1	153.7	MLP	2.0	3.0
		CNN	1.9	2.1
2	237.5	MLP	6.0	6.6
		CNN	1.8	3.9
3	328.7	MLP	6.7	7.0
		CNN	3.3	4.8
4	445.5	MLP	2.2	4.5
		CNN	1.5	3.3
5	537.9	MLP	5.0	5.3
		CNN	2.1	3.4
6	653.4	MLP	11.0	11.7
		CNN	2.6	4.7
7	726.9	MLP	6.0	8.0
		CNN	1.5	2.1
8	909.0	MLP	11.5	19.2
		CNN	1.4	1.5

**Table 3:** *RMSE* and *MAE* of I–V curve obtained by equivalent circuit method.

Working Conditions	1	2	3	4
**Irradiance *G* (W/m^2^)**	153.7	328.7	537.9	726.9
***MAE***	0.443	0.975	1.236	0.998
***RMSE***	0.335	0.766	0.895	0.864

**Table 4:** Comparison between artificial intelligence and equivalent circuit method.

Evaluation Method	Equivalent Circuit Method	CNN Model	MLP Model
***MAE***	0.864	0.0307	0.0671
***RMSE***	0.445	0.0322	0.0816
